# Concentration-dependent effects of boron fertilizer on growth, yield, and quality of buckwheat

**DOI:** 10.3389/fpls.2025.1548792

**Published:** 2025-08-04

**Authors:** Ying Jiang, Sunyu Wang, Yanmin Liu, An Wang, Lei Chang, Yingchun Cai, Tian Yu, QingTao Chang

**Affiliations:** ^1^ Characteristic Grain and Economy Research Laboratory, Taizhou Institute of Agricultural Science, Jiangsu Academy of Agricultural Sciences, Taizhou, Jiangsu, China; ^2^ Applied Meteorology, Nanjing University of Information Science and Technology, Nanjing, Jiangsu, China; ^3^ Miscellaneous Grains Room, Taixing Institute of Agricultural Sciences, Taixing, Jiangsu, China; ^4^ Inspection Laboratory, Taizhou Product Quality Supervision and Inspection Institute, Taizhou, Jiangsu, China

**Keywords:** buckwheat, boron fertilizer, seed germination, seedling growth, yield, quality

## Abstract

**Introduction:**

Boron is an essential trace element for plant growth and development, playing a critical role in flowering, fruit setting, nutrient transport, and stress resistance in crops. Buckwheat is‌ an important coarse grain crop, ‌and‌ its yield and quality are easily affected by boron nutritional status.

**Methods:**

In order to identify the optimal concentration of boron fertilizer to maximize growth and yield of buckwheat, pot experiments and field trials were carried out at different mass concentrations of boron fertilizer solution: 0, 12, 24, 48, and 72 mg/L.

**Results:**

The results showed that 48 mg/L boron fertilizer solution promoted seed germination and seedling growth of Suqiao 1, whereas 24 mg/L boron fertilizer solution had the best effect on seed germination and seedling growth of 1412-69. Moreover, the levels of activity of superoxide dismutase (SOD), catalase (CAT), and peroxidase (POD) of Suqiao 1 seedlings were highest under the 48 mg/L boron fertilizer treatment, and those in 1412–69 were highest under the 24 mg/L treatment. The highest grain yield and grain quality of Suqiao 1 were found at a boron concentration of 48 mg/L, whereas grain yield and quality of 1412–69 were maximized at a concentration of 24 mg/L.

**Discussion:**

In summary, boron fertilizer solutions at optimal concentrations can effectively stimulate buckwheat seed germination and seedling growth and thus enhance buckwheat yield.

## 1 Introduction

Buckwheat is a widely cultivated medicinal and edible crop. There are two main cultivated species: common buckwheat (*Fagopyrum esculentum* Moench) and Tartary buckwheat (*Fagopyrum tataricum* Gaertn.). There is a long history of cultivation in China. In northern China, sweet buckwheat is mainly grown, while in southern China, bitter buckwheat is the main crop (Kreft et al., 2022a; Tomasiak et al., 2022; Zargar et al., 2024). The advantageous characteristics of buckwheat include short growth cycle, resistance to disease and insect pests, and adaptability to varied environments. The nutritional components of buckwheat include balanced amino acids, dietary fiber, rutin, and various vitamins and minerals (Skřivan et al., 2023; Kumar et al., 2024). In recent years, with the improvement in living standards, the nutritional value of buckwheat has been widely valued, and the demand for buckwheat has increased, bringing good opportunities for the development of the buckwheat industry.

Buckwheat, a crop suitable for cultivation under harsh ecological conditions, is used as a raw material for cooking in Europe, the United States, and Asia (Singh et al., 2020). As a nutrient-rich raw food, buckwheat is rich in protein and the essential amino acid lysine. Food made from buckwheat can prevent many human diseases, such as diabetes, cardiovascular disease, hypertension and cancer (Kurćubić et al., 2024; Li et al., 2025). In addition, buckwheat grain contains bioactive compounds with potential antiviral effects, such as rutin, quercetin, and emodin, which make buckwheat highly nutritious (Huda et al., 2021). Rutin is a flavonoid that has been shown to have a wide range of health promoting effects, such as antibacterial, anti-inflammatory, and anti-cancer properties. Under humid conditions, most rutin in buckwheat is degraded to quercetin by rutin-degrading enzymes (rutinases) (Wang et al., 2024a). Furthermore, quercetin has been widely studied because of its various beneficial effects on human health, including antioxidant, free radical-scavenging, anti-inflammatory, cardioprotective, hepatoprotective, and antibacterial properties (Mirza et al., 2023).

Crop growth requires many nutrients, and it is difficult to achieve high yields based solely on the nutrients in the soil. The growth period of buckwheat is short; buckwheat plants grow rapidly during the vegetative growth period and require large amounts of nutrients. Therefore, fertilization is a key factor affecting the yield and quality of buckwheat (Wan et al., 2023). A previous study found that the combined application of chemical fertilizers, organic fertilizers, and biochar could significantly increase the resistant starch content, amylose content, solubility, swelling capacity, and light transmittance of buckwheat starch, which are factors that improve the quality of common buckwheat (Tao et al., 2023a). Appropriate nitrogen (N) fertilizer could effectively improve endosperm development, starch synthesis and accumulation, and grain traits of common buckwheat (Gao et al., 2023). Another study showed that phosphorus (P) fertilizer could affect the growth and quality of Tartary buckwheat (Zhang et al., 2020). In conclusion, fertilization plays an important role in improving buckwheat yield and grain quality.

Boron, an essential nutrient for plant growth, plays an indispensable role in the structure and stability of plant cell walls and membranes, synthesis and transport of nutrients and water, photosynthesis, and metabolism of proteins and nucleic acids (Qu et al., 2024). The symptoms of boron deficiency are diverse and can have a severe impact on root and shoot development, thereby limiting crop yield and quality (Chu et al., 2025). Boron is absorbed mainly in the form of boric acid through the roots, and root growth is more sensitive than shoot growth to boron deficiency. Boron deficiency inhibits the growth of new tissues and inhibits or stops the growth of root and shoot tips. When boron is seriously deficient, the top buds stop growing, gradually wither, and die; the roots are underdeveloped; the leaves are dark green; the leaves become small, thickened, and shrunken; the flowers are not well developed; the buds are all shed; the flowering period is prolonged; the grain ears are not fruiting; and the roots and berries decay or become necrotic (Chu et al., 2025). Boron fertilizer is applied in the production of rice, wheat, soybean, and oilseed rape (Wang et al., 2022; Traspadini et al., 2023; Pachauri et al., 2024; Xie et al., 2024). Boron deficiency is prevalent in the soils of Gaogang District, Jiangsu Province, particularly in sandy soils, where boron is prone to leaching, resulting in effective boron levels below the crop demand threshold. In boron-deficient environments, buckwheat exhibits impaired root development and reduced photosynthetic efficiency. The application of exogenous boron fertilizer can significantly enhance plant stress resistance and yield potential. The ubiquity of boron deficiency in Jiangsu soils, coupled with the irreplaceable role of boron in the growth and development of buckwheat, underscores the need for boron fertilizer application. However, the role of boron fertilizer in buckwheat has rarely been studied.

To address these gaps in the literature, we used different concentrations of boron fertilizer solutions to treat two varieties of buckwheat, Suqiao 1 and 1412-69, and analyzed their effects on seed germination, seedling growth, physiological indicators, yield, and grain quality. The results provide a more solid theoretical basis and practical guidance for the scientific and rational application of boron fertilizer in buckwheat production.

## 2 Materials and methods

### 2.1 Test material

The tested buckwheat varieties were Suqiao 1 and 1412-69, a total of two buckwheat varieties. The seeds of Suqiao 1 and 1412–69 used in this study were collected from Taizhou Institute of Agricultural Sciences, Jiangsu Academy of Agricultural Sciences. Suqiao 1 was certified as a provincial crop variety by Jiangsu Province variety in 2015 (Identification No.: Su Buckwheat 201501). Developed through hybridization with the local Taixing buckwheat variety by the Taizhou Agricultural Science Institute of Jiangsu Academy of Agricultural Sciences, this cultivar demonstrates high yield and strong stress resistance, making it suitable for cultivation throughout Jiangsu’s buckwheat production regions. Meanwhile, 1412–69 is a novel sweet buckwheat variety bred by the same institute. In 2024, it was submitted for both Jiangsu provincial variety certification and national plant variety rights protection, with similar regional adaptability.

### 2.2 Overview of the experimental area

The experiment was conducted in 2021 in Gaogang District, Jiangsu Province, China. The experimental site is located in central Jiangsu Province on the north bank of the Yangtze River (32°01’~33°10’ N, 119°38’~120°33’ E), with an altitude of 5-7m. The area lies in the middle-lower Yangtze River basin, characterized by a subtropical monsoon climate with distinct seasons, an annual average temperature of 17.4 °C, and abundant rainfall (1,281 mm/year) with a 220-day frost-free period. The experimental field has high sandy soil containing 10.0g/kg organic matter, 4.5mg/kg available phosphorus, and 57mg/kg available potassium in the topsoil. Soil available boron in Gaogang District ranges 0.2-0.5 mg/kg - below the 0.5-1.0 mg/kg threshold required for healthy buckwheat growth, indicating boron deficiency. Therefore, five boron fertilizer treatments (0, 12, 24, 48, and 72 mg/L) were applied to determine the optimal concentration, covering the range from deficiency to sufficiency levels.

### 2.3 Germination test

The buckwheat seeds were rinsed with distilled water to remove impurities, and plump seeds with uniform size and no defect were selected. Selected seeds were soaked for 8 hours in boron fertilizer solution (0, 12, 24, 48, and 72 mg/L), while the control group received distilled water. Subsequently, seeds were washed with distilled water, surface-dried, and evenly place them in 90 mm Petri dishes lined with filter paper. Thirty seeds were allocated per dish, with 3 mL of corresponding boron fertilizer solution added via pipette for room-temperature cultivation. Each treatment was replicated five times. To maintain filter paper saturation, experimental groups received daily supplementation of their respective boron solutions, whereas controls received distilled water.

### 2.4 Pot experiment

**Figure 1:**
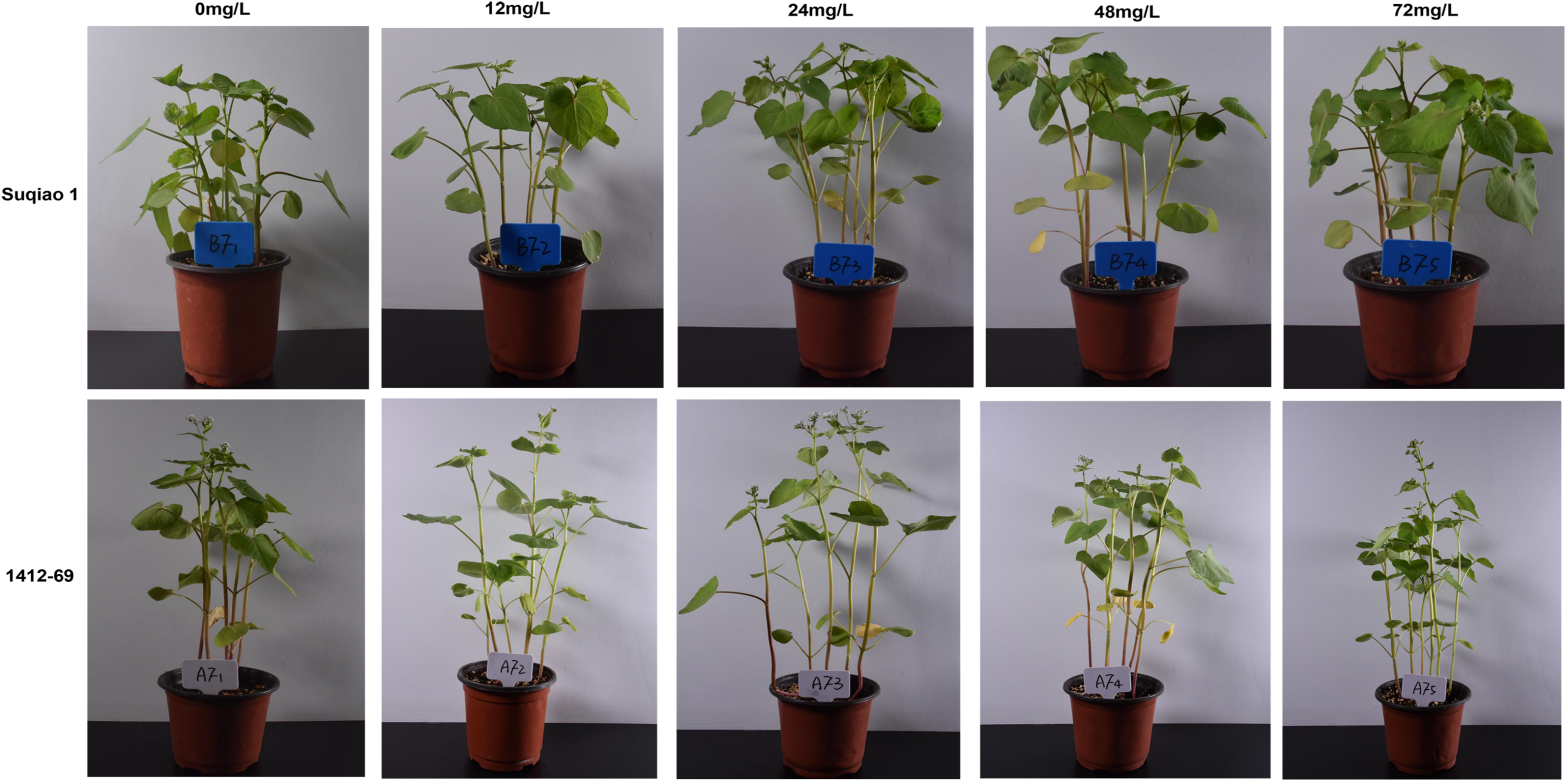
Morphological changes of Suqiao 1 and 1412–69 under Different Boron Fertilizer Concentrations. From left to right, the morphological changes of two buckwheat varieties (Suqiao 1 and 1412-69) under different boron fertilizer solution concentrations (0, 12, 24, 48, and 72 mg/L) are represented successively. The results show that with the increase in the concentration of the boron fertilizer solution, the growth status of the two buckwheat varieties has changed significantly. When the concentration of the boron solution is 48 mg/L, Suqiao 1 grows the best. When the concentration of the boron fertilizer solution is 24 mg/L, 1412–69 grows the best. It can be found that there are differences in the sensitivity of Suqiao 1 and 1412–69 to the concentration of the boron fertilizer.

The potted plant experiment was conducted in a solar greenhouse. Tested buckwheat seeds were cultured in plastic flowerpots (10 cm radius × 25 cm depth) with five seeds per pot. Each pot contained 18 kg of air-dried soil, with nitrogen (0.54 g/kg), phosphorus (1.06 g/kg), and potassium fertilizer (0.26 g/kg) applied as base fertilizers. At the seedling stage, 100 mL of boron solution (varying concentrations) was applied, while the control group received distilled water. Both Suqiao 1 and 1412–69 received identical treatment. The experiment comprised six treatments with six replicates each (36 pots per variety). After five days, various indices were measured. **Figure 1** illustrates the morphological changes in both varieties.

### 2.5 Field trial

In this experiment, five treatments were established with boron solution concentrations of 0, 12, 24, 48, 72 mg/L respectively, plus a boron-free control (CK). Each treatment was replicated three times. During the experiment, the watering amount was determined based on the water shortage situation of buckwheat at each growth stage, and deionized water was used for watering. Experimental plots (10 m² = 2 × 5 m) were drill-sown with uniform 33 cm row spacing. Suqiao 1 and 1412–69 received 45 g and 60 g sowing rates respectively under conventional management. During budding stage, single foliar sprays were applied in calm conditions using concentration-specific boron solutions (450 L/hm² spray volume), ensuring leaf wetness without runoff. Before and after the experiment, the soil’s available boron content was measured to ascertain the soil’s boron supply capacity, providing a safe threshold reference for long-term application.

### 2.6 Determination index and method

The germination of the seeds was recorded at the same time every day (as long as the plumule length reached the seed length). Germination potential was measured on the 3rd day of germination, germination rate, root length, bud length and fresh weight were measured on the 7th day. On the 7th day, the radicle and plumule of buckwheat seeds were mixed and stored at -80 °C for subsequent indexes determination. The germination rate, germination potential and vigor index were calculated. Fifteen plants were randomly selected for each treatment, and the average value was taken and repeated three times.


Seed germination potential (%) = Number of 3d germinated seeds/Seed count;

Seed germination rate (%) = 7d Number of germination seeds/Total number of seeds;

Germination index =∑Gt/Dt.

Where: Gt is the number of sprouting in time t (unit: d); Dt is the corresponding germinating days.


Vigor index = germination index × fresh weight of seedlings

After 5 days of seedling treatment, 15 representative plants were randomly selected and their plant height, stem diameter (measure the diameter of the stem base with a vernier caliper), fresh weight, number of main stem segments, number of main stem branches and number of leaves per plant were measured. Finally, the average was taken and repeated three times. The chlorophyll Soil and Plant Analyzer Development (SPAD) value was determined by SPAD-502 chlorophyll meter produced in Japan. Fifteen representative plants were selected and the leaves of the same part were measured. Three points on each leaf were measured and the average value was calculated. Grain number per plant, grain weight per plant, thousand grain weight, and plot yield of buckwheat were measured at maturity.

Physiological index determination: Superoxide dismutase (SOD) activity was determined by nitrogen blue tetrazole colorimetric method (NBT). The activities of peroxidase (POD) and catalase (CAT) activity were analyzed by Ultraviolet (UV) absorption method, and the content of malondialdehyde (MDA) was determined by thiobarbituric acid (TBA) color development method.

Quality index measurement: The grain protein content was detected by Coomassie brilliant blue G-250 method, and the grain starch content was analyzed by anthrone colorimetry. The fat content of grain was evaluated by near infrared spectroscopy, and the total flavonoids of grain was determined by spectrophotometry. The oil was extracted from seeds and the content of rutin and quercetin in the oil was determined.

### 2.7 Gene expression analysis

Total RNA was extracted from frozen samples (roots, stems, leaves, and seeds) using TRIzol reagent (Takara, Beijing, China). Following the manufacturer’s instructions, the RNA was reverse transcribed into cDNA using a cDNA synthesis kit (gDNA Purge, Novoprotein, China). Quantitative RT-PCR was performed using SYBR Green chemistry on a Roche LightCycler^®^ 480 instrument. CACS serves as the internal reference gene of Suqiao1 (Demidenko et al., 2011), and H3 serves as the internal reference gene of 1412-69 (Yao et al., 2022). PCR amplification data were analyzed using the 2^−△△CT^ method, with three experimental replicates for reproducibility.

### 2.8 Statistical analysis

Data were organized and graphed using Microsoft Excel 2016 and GraphPad Prism 7.0, respectively. Prior to analysis of variance, all data were examined for normality using the Shapiro-Wilk test and for homogeneity of variance using Levene’s test. If the data exhibited a normal distribution (*P* ≥ 0.05) and homogeneous variances (*P* ≥ 0.05), one-way ANOVA followed by Duncan’s multiple range test (α = 0.05) was performed. When homogeneity of variances was not satisfied, Welch’s corrected ANOVA was applied. For non-normally distributed data, the Kruskal-Wallis non-parametric test was utilized. All statistical analyses were performed using SPSS 22.0. All statistical significance was based on the threshold of *P*<0.05.

## 3 Results

### 3.1 Effects of boron fertilizer application at different mass concentrations on buckwheat seed germination

**Figure 2:**
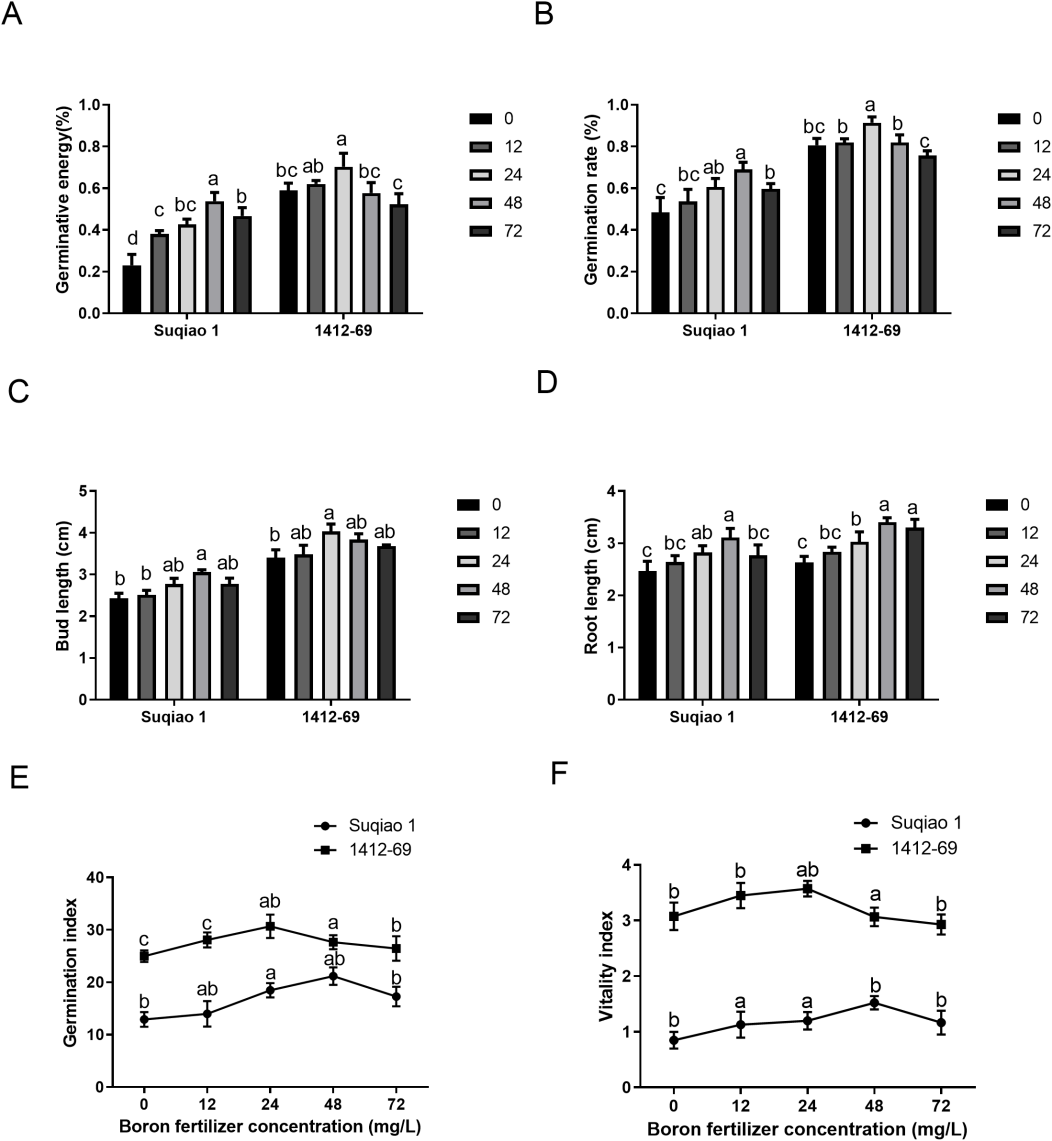
Effect of boron fertilizer solution with different mass concentration on germination of buckwheat seeds. **(A)** Effect of boron fertilizer solution with different mass concentration on germination potential of buckwheat seeds. **(B)** Effect of boron fertilizer solution with different mass concentration on germination rate of buckwheat seeds. **(C)** Effect of boron fertilizer solution with different mass concentration on germination length of buckwheat seeds. **(D)** Effect of boron fertilizer solution with different mass concentration on root length of buckwheat seeds. **(E)** Effect of boron fertilizer solution with different mass concentration on germination index of buckwheat seeds. **(F)** Effect of boron fertilizer solution with different mass concentration on vigor index of buckwheat seeds. Bars superscripted by different lowercase letters are significantly different at the 0.05 probability level (the same below).

All the measured growth parameters (germination potential, germination rate, bud length, root length, germination index, and vitality index) of both buckwheat varieties exhibited bell-shaped response curves (**Figure 2**). In general, seed germination potential, germination rate, bud length, root length, germination index, and vitality index of the 1412–69 variety were better than those of Suqiao 1. It is worth noting that the seed germination potential and germination rate of Suqiao 1 were highest under treatment with 48 mg/L boron fertilizer solution, with values of 53.7% and 68.9%, respectively, representing increases of 133.78% and 43.22%, respectively, compared to CK (*P*<0.05). The germination potential and rate of seed germination were highest in the 1412–69 plants treated with 24 mg/L boron fertilizer solution, up to 70.33% and 91.4%, respectively; these values represent an increase of 19.20% and 13.51% compared to the corresponding values in the CK group (*P*<0.05). However, seed germination was inhibited when the concentration of boron fertilizer solution was too high (**Figures 2A, B**). In addition, the bud length and root length of Suqiao 1 were highest (3.06 cm and 3.11 cm, respectively) when the mass concentration of boron fertilizer solution was 48 mg/L, and these results represent a significant increase of 25.93% and 25.91%, respectively (*P*<0.05), compared to the CK group. The bud growth of 1412–69 was highest (4.03 cm) when the mass concentration of boron solution was 24 mg/L, and this result is 18.53% higher than that of the CK, respectively (*P*<0.05). The root length was highest (3.3 cm) when the mass concentration of boron solution was 48 mg/L, and this result is a significant increase (25.48%, *P*<0.05) compared to the CK group. When the concentration of boron fertilizer solution was too high, bud and root growth were inhibited (**Figures 2C, D**). In the case of the Suqiao 1 plants, the germination and vitality indices were highest (21.19 and 1.52, respectively) at 48 mg/L boron, and these results represented a significant increase, 63.88% and 78.82%, respectively, compared to the CK group. The germination and vitality index of 1412–69 reached their highest values at 24 mg/L, which were 30.67 and 3.57, representing an increase of 25.23% and 16.29%, respectively, compared with the control group. Similarly, when the concentration of boron fertilizer solution was too high, the germination and vitality indices were inhibited (**Figures 2E, F**).

### 3.2 Effects of boron fertilizer solution at different mass concentrations on growth of buckwheat seedlings

**Figure 3:**
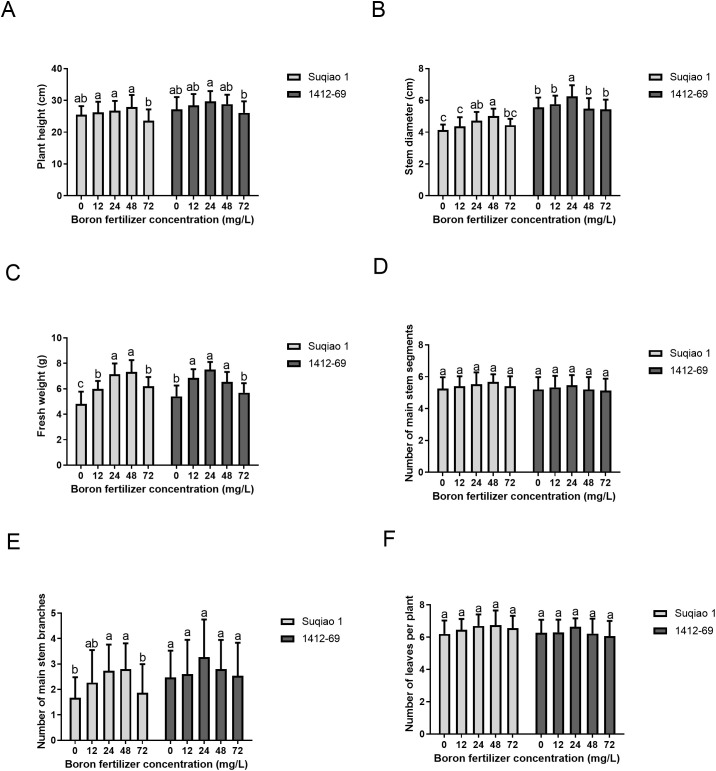
Effects of boron fertilizer solution with different mass concentration on growth of buckwheat seedlings. **(A)** Effect of different concentrations of boron fertilizer solution on buckwheat plant height. **(B)** Effect of different concentrations of boron fertilizer solution on stem diameter of buckwheat. **(C)** Effect of different concentrations of boron fertilizer solution on fresh weight of buckwheat **(D)** Effect of different concentrations of boron fertilizer solution on the number of nodes of buckwheat main stem. **(E)** Effects of different concentrations of boron fertilizer solution on the number of branches of buckwheat main stem. **(F)** Effects of different concentrations of boron fertilizer solution on the number of leaves per main buckwheat plant.

As shown in **Figure 3**, growth parameters—plant height, stem diameter, fresh weight, number of main stem segments, branches of the main stem, and number of leaves per plant—of the two buckwheat varieties first increased and then decreased with an increase in the mass concentration of the boron fertilizer solution, and the effect of the boron fertilizer solution on 1412–69 seedlings was greater than that on Suqiao 1 seedlings. When the mass concentration of boron fertilizer solution was 48 mg/L, the plant height, stem diameter, and fresh weight per plant of Suqiao 1 reached their highest values, 27.95 cm, 5.02 cm and 7.32 g respectively. These results are significantly higher (9.35%, 21.14%, and 52.18%, respectively (*P*<0.05), compared with CK. When the mass concentration of the boron solution was 24 mg/L in 1412-69, plant height, stem diameter, and fresh weight per plant reached their highest values: 29.72 cm, 6.27 cm and 7.51 g, respectively. Compared to CK, these results represent a significant increase, 9.30%, 12.77%, and 39.07%, respectively. A high concentration of boron fertilizer was not conducive to the growth of Suqiao 1 and 1412–69 seedlings (**Figures 3A-C**). In addition, the number of main stem segments, number of main stem branches, and number of leaves per plant in Suqiao 1 reached their highest values when the mass concentration of boron solution was 48 mg/L; the average values were 5.67, 2.80, and 6.76, respectively, which are increases of 7.59%, 67.66%, and 9.21%, respectively, compared with the CK. However, the number of main stem segments, main stem branches, and leaves per plant of 1412–69 reached their highest values when the mass concentration of the boron solution was 24 mg/L; the average values were 5.46, 3.26, and 6.64, respectively, which represent increases of 5.00%, 31.38%, and 6.07%, respectively, compared with the CK group. Although the buckwheat plants were in the seedling stage at this time, boron solution treatment with different mass concentrations still affected the number of main stem segments, the number of main stem branches, and the number of leaves per plant (**Figures 3D-F**).

**Figure 4:**
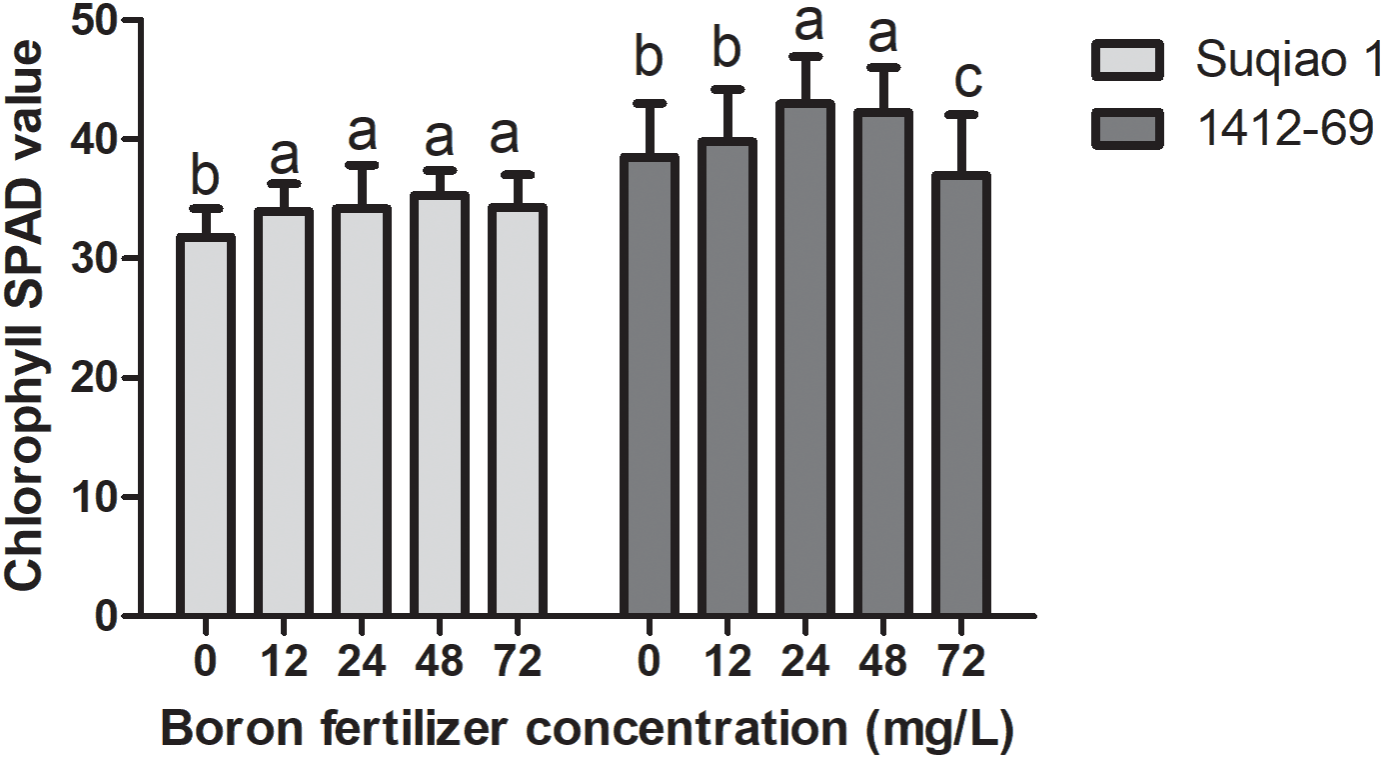
Effect of boron fertilizer solution with different mass concentration on chlorophyll SPAD value of buckwheat seedlings.

The effects of boron fertilizer solution at different mass concentrations on chlorophyll SPAD values of the buckwheat seedlings are shown in **Figure 4**. It can be seen from the figure that chlorophyll SPAD initially increased and then decreased with the increase in boron fertilizer solution mass concentration. The chlorophyll SPAD value of Suqiao 1 was highest when the mass concentration of the boron solution was 48 mg/L, and this result is a 10.94% increase over the values obtained in the CK group (*P*<0.05). The chlorophyll SPAD value of 1412–69 was highest when the mass concentration of the boron fertilizer solution was 24 mg/L; this result represents an increase of 13.34% compared with the results in the CK group (*P*<0.05). It can be seen that the change range of chlorophyll SPAD value in 1412–69 was greater than that in Suqiao 1, and the chlorophyll SPAD value in 1412–69 was more sensitive to the response to boron fertilizer solution.

### 3.3 Effects of boron fertilizer solution at different mass concentrations on physiological indexes of buckwheat

**Figure 5:**
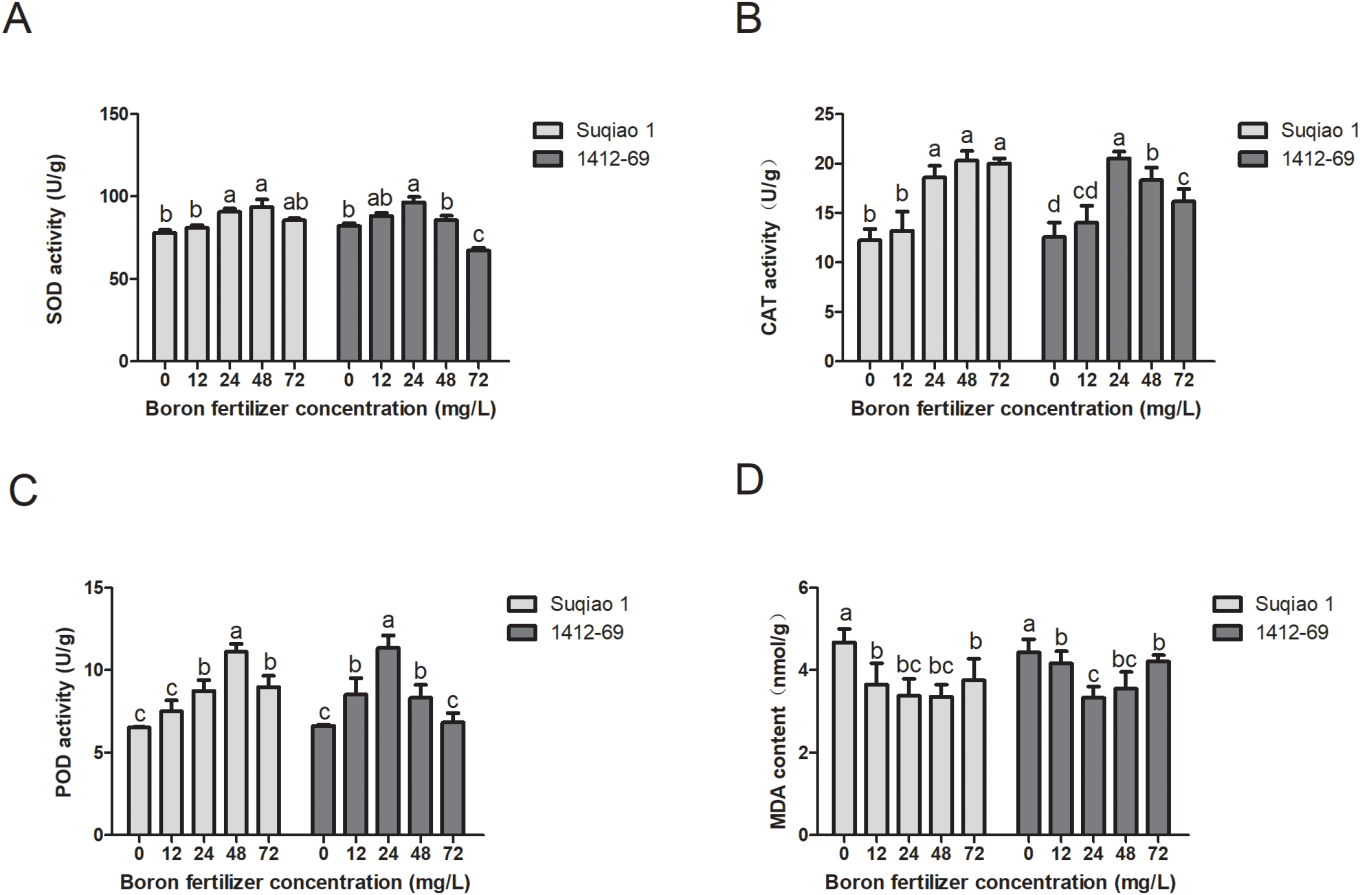
Effects of boron fertilizer solution with different mass concentrations on physiological indexes of buckwheat. **(A)** The effects of different concentrations of boron fertilizer solution on SOD activity of buckwheat seedlings. **(B)** Effects of different concentrations of boron fertilizer solution on CAT activity of buckwheat seedlings. **(C)** Effects of different concentrations of boron fertilizer solution on POD activity of buckwheat seedlings. **(D)** Effects of different concentrations of boron fertilizer solution on MDA content of buckwheat seedlings.

With an increase in the mass concentration of the boron fertilizer solution, the SOD activity of seedlings of the two buckwheat varieties first increased and then decreased, and the SOD activity of seedlings treated with each concentration was higher than that of the control group. When the concentration of the boron solution was 48 mg/L, the SOD activity of Suqiao 1 seedlings reached a maximum (93.72 U/g), which is a 20.08% increase (*P*<0.05) compared to the results in the CK group. When the mass concentration of boron fertilizer solution was 24 mg/L, the SOD activity of 1412–69 seedlings reached a maximum of (96.44 U/g), a 17.58% increase compared to CK (*P*<0.05). The results suggest that an appropriate concentration of boron fertilizer can improve the ability of SOD to scavenge oxygen free radicals in seedlings (**Figure 5A**). In addition, with an increase in boron concentration, the CAT activity of seedlings of both buckwheat varieties first increased and then decreased. When the mass concentration of the boron solution was 48 mg/L, the CAT activity of Suqiao 1 seedlings was the highest, 39.53% higher than that of the CK (*P*<0.05). When the mass concentration of the boron solution was 24 mg/L, the CAT activity of 1412–69 seedlings reached a maximum value that was 39.16% higher than that of the CK (*P*<0.05). The results show that an appropriate concentration of boron fertilizer can increase the CAT activity of seedlings (**Figure 5B**). Moreover, the effects of boron solution on the POD activity of the two buckwheat varieties differed depending on concentration; enzymatic activity first increased and then decreased with increasing concentration of boron, although the POD activity of each experimental group (each boron concentration) was higher than that of the CK group. When the mass concentration of the boron fertilizer solution was 48 mg/L, the POD activity of Suqiao 1 seedlings was highest, increasing by 41.31% compared with that of CK (*P*<0.05). In the case of the 1412–69 seedlings, maximum POD activity (41.55% higher than that of CK, *P*<0.05) was obtained when the boron solution was 24 mg/L (**Figure 5C**).

With respect to MDA content, the negative effect of boron treatment was evident. The MDA content of seedlings of the two buckwheat varieties first decreased and then increased with increasing boron concentration. This result is contrary to the trend of SOD, CAT, and POD, and the MDA content of all treatment groups was lower than that of the CK group. The MDA content of Suqiao 1 seedlings was lowest, at 4.67 nmol/g, a 28.05% decrease compared to that of CK (*P*<0.05), when the mass concentration of the boron solution was 48 mg/L. In the case of the 1412–69 seedlings, the MDA content was lowest, at 4.30 nmol/g, a 22.56% decrease compared to CK (*P*<0.05) when the concentration of boron solution was 24 mg/L (**Figure 5D**).

### 3.4 Influence of boron fertilizer solution at different mass concentration on buckwheat yield

As shown in **Table 1**, the yield of two buckwheat varieties at first increased and then decreased with increasing concentration of boron fertilizer solution. With respect to Suqiao 1 plants, measures of crop yield compared to CK results—number of grains per plant (13.62%), grain weight per plant (10.49%), thousand grain weight (11.20%), and plot yield (18.63%)—were highest when the concentration of boron was 48 mg/L; these increases were found to be significant (*P*<0.05). In the case of the 1412–69 seedlings, a boron concentration of 24 mg/L produced the highest increases in grains per plant (16.03% increase compared to CK), grain weight per plant (20.25%), thousand grain weight (10.33%), and plot yield (14.55%); these differences were found to be statistically significant (*P*<0.05). Thus, by comparing the plot yield of two buckwheat varieties under different concentrations of boron fertilizer application, we found that application of 48 mg/L boron fertilizer can effectively increase the yield of Suqiao 1, and application of 24 mg/L boron fertilizer can effectively increase the yield of 1412-69.

**Table 1:** Influence of boron fertilizer solution with different mass concentration on yield of buckwheat.

Variety	Treatment (mg/L)	Grain number per plant/grain	Grain weight per plant/g	Thousand grain weight/g	Plot yield/kg
Suqiao1	0	152.34 ± 2.74c	2.67 ± 0.21b	19.19 ± 0.39bc	1.02 ± 0.08b
	12	164.25 ± 3.25b	2.81 ± 0.17ab	20.36 ± 0.91b	1.18 ± 0.04a
	24	166.27 ± 4.35b	2.83 ± 0.23ab	20.30 ± 1.09b	1.20 ± 0.03a
	48	173.36 ± 3.87a	2.95 ± 0.20a	21.34 ± 1.65a	1.21 ± 0.04a
	72	163.36 ± 4.67b	2.76 ± 0.22b	20.13 ± 0.75b	1.19 ± 0.14a
1412-69	0	158.41 ± 3.24c	3.21 ± 0.19c	28.26 ± 0.57b	1.15 ± 0.13b
	12	167.37 ± 4.02b	3.61 ± 0.20b	30.55 ± 0.53b	1.17 ± 0.10b
	24	183.80 ± 5.25a	3.86 ± 0.21a	31.18 ± 0.82a	1.31 ± 0.05a
	48	175.09 ± 3.45b	3.65 ± 0.23b	30.85 ± 0.82b	1.17 ± 0.06b
	72	165.36 ± 3.67b	3.52 ± 0.22b	30.52 ± 1.33b	1.02 ± 0.05c

### 3.5 Influence of boron fertilizer solution at different mass concentrations on buckwheat grain quality

**Figure 6:**
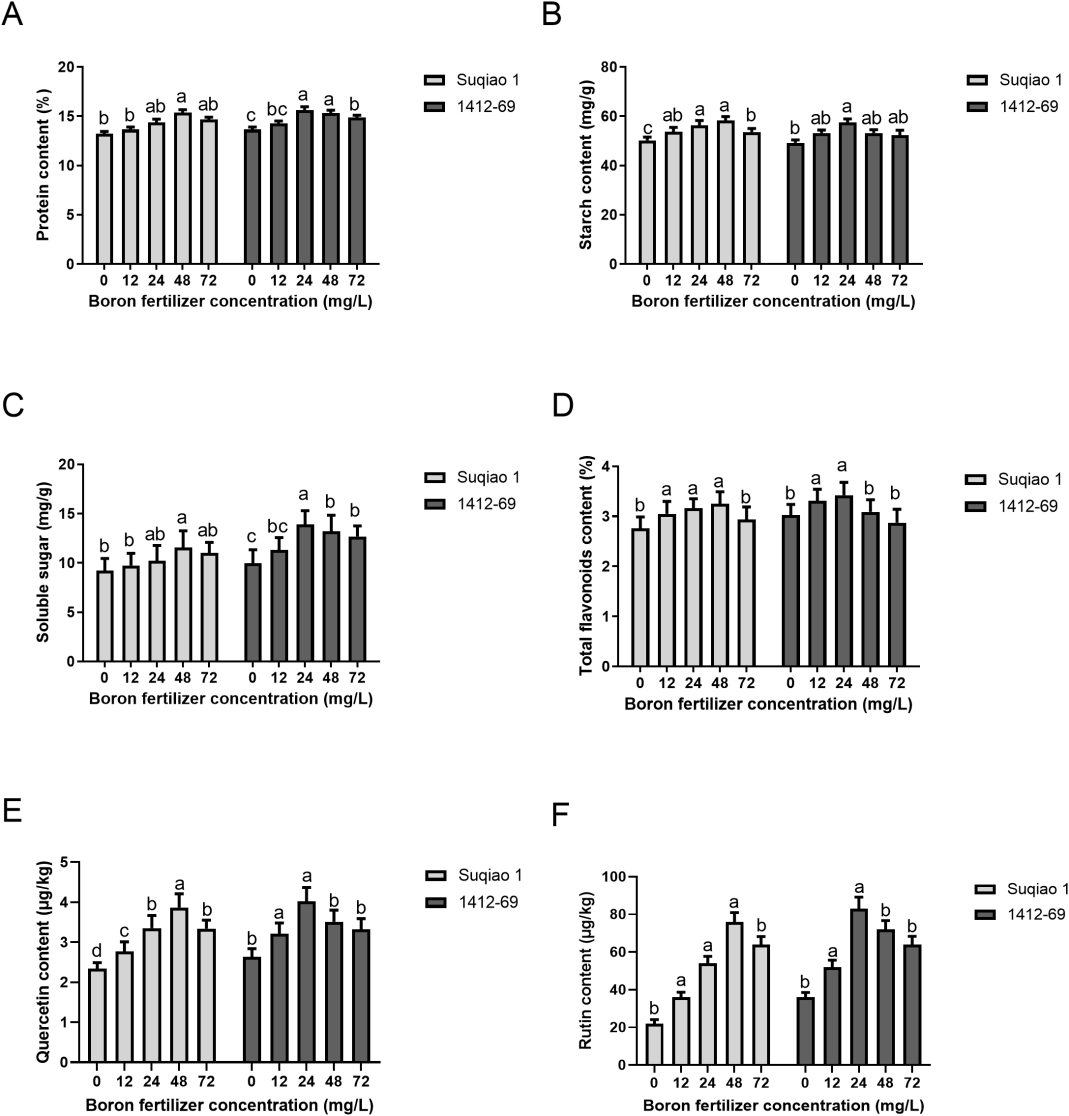
Effects of different concentrations of boron fertilizer solution on buckwheat grain quality. **(A)** Effects of different concentrations of boron fertilizer solution on buckwheat grain protein. **(B)** Effects of different concentrations of boron fertilizer solution on buckwheat grain starch. **(C)** Effects of different concentrations of boron fertilizer solution on buckwheat grain fat. **(D)** Effects of different concentrations of boron fertilizer solution on total flavonoids in buckwheat grains. **(E)** Effects of different concentrations of boron fertilizer solution on quercetin content in buckwheat grains. **(F)** Effects of different concentrations of boron fertilizer solution on the rutin content in buckwheat grains. Data are presented as mean ± standard error of the mean. Bars superscripted by different lowercase letters are significantly different at the 0.05 probability level.

As shown in **Figure 6**, the effect of boron fertilizer on buckwheat grain quality depends on concentration. With the increase in boron fertilizer concentration, the contents of protein, starch, soluble sugar, total flavonoids, quercetin and rutin in the two buckwheat varieties initially increased and then decreased (**Figures 6A-F**). At a boron concentration of 48 mg/L, the contents of protein, starch, soluble sugar, total flavonoids, quercetin, and rutin in Suqiao 1 were 15.36%, 58.23 mg/g, 11.56 mg/g, 3.25%, 3.84 μg/kg, and 76.11 μg/kg respectively. These results represent significant (*P*<0.05) increases—16.10%, 15.97%, 25.24%, 17.75%, 63.40%, and 246.58%— compared with the CK group. When the mass concentration of boron fertilizer solution was 24 mg/L, the content of protein, starch, soluble sugars, total flavonoids, quercetin and rutin in 1412–69 buckwheat grains all reached their maximum values, at 15.62%, 57.59 mg/g, 13.93 mg/g, 3.42%, 4.02 μg/kg, and 81.87 μg/kg, respectively, and these values represent significant (*P*<0.05) increases of 14.43%, 16.98%, 39.58%, 12.87%, 52.27%, and 125.48%, respectively, compared to the corresponding results in the CK group.

**Figure 7:**
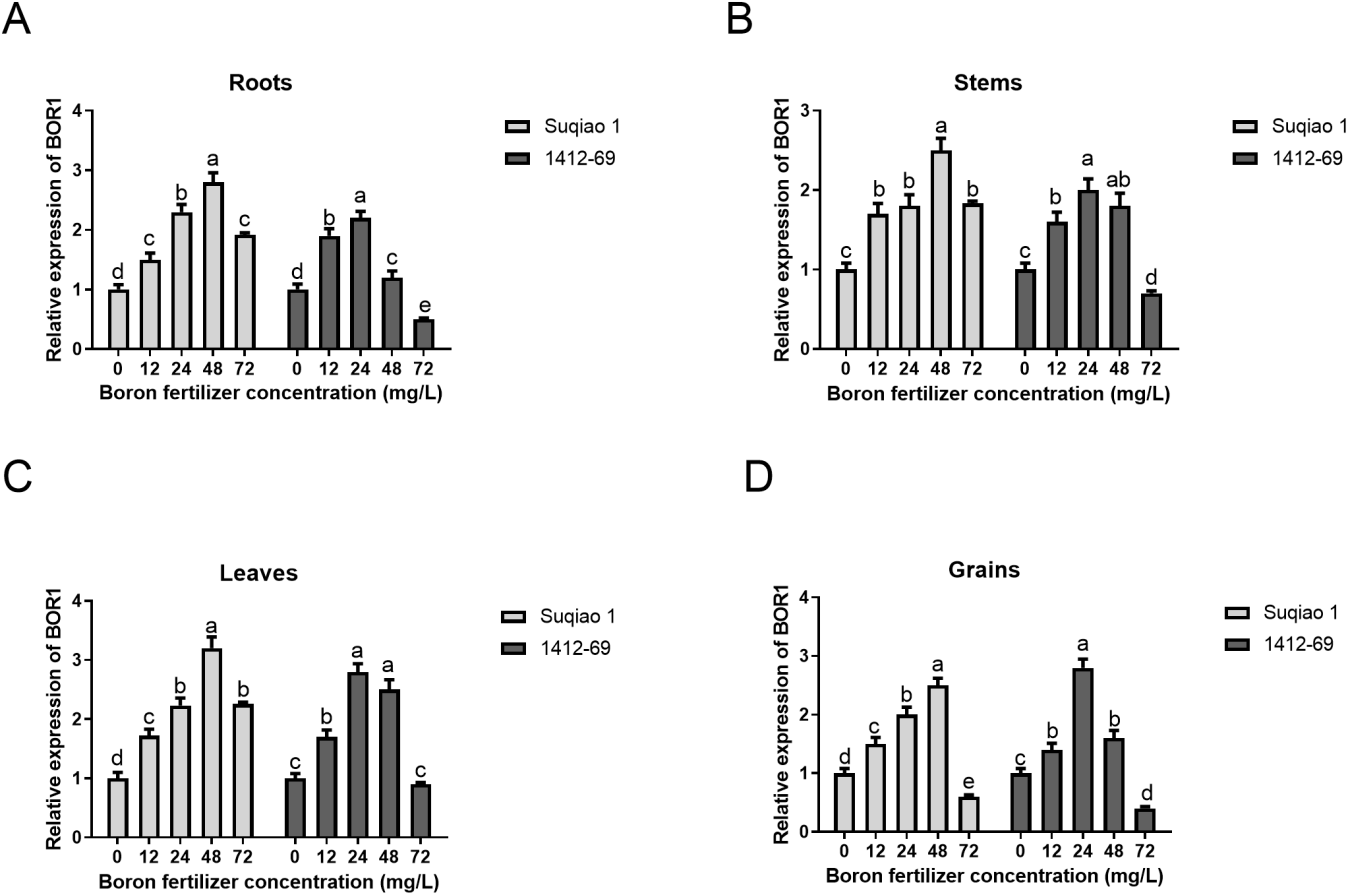
Effects of boron fertilizer solutions at different concentrations on the expression of boron transporter BOR1 in buckwheat. **(A)** Effects of boron fertilizer solutions at different concentrations on BOR1 expression in buckwheat roots. **(B)** Effects of boron fertilizer solutions at different concentrations on BOR1 expression in buckwheat stems. **(C)** Effects of boron fertilizer solutions at different concentrations on BOR1 expression in buckwheat leaves. **(D)** Effects of boron fertilizer solutions at different concentrations on BOR1 expression in buckwheat grains. Data are presented as mean ± standard error of the mean. Bars superscripted by different lowercase letters are significantly different at the 0.05 probability level.

Additionally, we examined the expression levels of BOR1 in frozen samples (roots, stems, leaves, and grains) of mature buckwheat treated with boron fertilizer at different concentrations. The results indicated that, as boron concentration increased from 0 to 48 mg/L in Suqiao 1, the expression of BOR1, a boron transporter protein, was significantly upregulated in roots, stems, leaves, and grains, reaching a peak at a concentration of 48 mg/L. When the boron concentration exceeded 48 mg/L, BOR1 expression showed feedback inhibition (**Figures 7A-D**). In contrast, in 1412–69 plants, as boron concentration increased from 0 to 24 mg/L, BOR1 expression was significantly upregulated in roots, stems, leaves and grains, peaking at a concentration of 24 mg/L. When the boron concentration surpassed 24 mg/L, BOR1 expression similarly showed feedback inhibition, suggesting a weaker tolerance to boron toxicity in this variety (**Figures 7A-D**).

## 4 Discussion

### 4.1 Concentration-dependent effect of boron fertilizer on the growth and development of buckwheat

As unique reproductive organs, seeds are a crucial component of plant production. Early emergence and uniform and strong seedlings are beneficial for increasing crop yield and producer income. In conventional agricultural operations, external environmental factors, such as light conditions, temperature, and soil moisture during sowing, as well as internal factors, such as seed vitality and endogenous hormone levels, can significantly influence seed germination. Determining the optimal conditions for seed germination is a key factor in seed reproduction, and can provide a scientific basis for seed expansion (Ali et al., 2022; Zhang et al., 2024). In the present study, with increasing boron fertilizer concentration, the germination potential, germination rate, bud length, root length, germination index, and vitality index of Suqiao 1 and 1412–69 seeds all showed a trend of first increasing and then decreasing. This may be related to the stabilizing effect of boron on the chlorophyll structure, promoting carbohydrate synthesis and transportation. An appropriate concentration of boron fertilizer solution can significantly improve the germination potential, germination rate, shoot length, root length, germination index, and vitality index of buckwheat seeds, but an excessively high concentration of boron inhibits the germination of buckwheat seeds, as was also found in the case of jute seeds in a previous study (Kumar et al., 2025). Boron deficiency has been reported to inhibit the growth of wild-type *Arabidopsis* seedlings (Tao et al., 2023b). This was mainly because boron deficiency increased the content of auxin in the root tips, leading to root growth inhibition. In another study (Rehman et al., 2022), the authors reported that the germination time of mung bean (*Vigna radiata* L.) seeds treated with 0.01% boron was significantly shortened, and growth indicators, namely average emergence time, emergence index, root length, plant height, and chlorophyll content were significantly improved. The authors found that both boron deficiency (hydroponics) and boron excess (over 0.01%) are detrimental to the growth and productivity of mung beans, which is similar to the findings we report here: plant height, stem diameter, fresh weight, number of main stem nodes, number of main stem branches, and number of leaves per plant of Suqiao 1 and 1412–69 seedlings showed a trend of first increasing and then decreasing with increasing boron fertilizer concentration. Moreover, the effect of boron fertilizer on 1412–69 seedlings was greater than that on Suqiao 1 seedlings. Growth indicators of Suqiao 1 reached their maximum values when the mass concentration of boron fertilizer solution was 48 mg/L; the growth indicators of 1412–69 seedlings were highest when the concentration of boron was 24 mg/L, indicating that boron can enter the buckwheat plants through leaf stomata, promoting the synthesis and stability of buckwheat cell wall and membrane components, and indirectly regulating the metabolism of auxin and lignin, which is helpful for the growth of buckwheat seedlings. However, excessive concentration of boron fertilizer is not conducive to the growth of the buckwheat varieties in the present study: Suqiao 1 and 1412-69. We speculated that boron could enter buckwheat through the stomata of leaves and promote the synthesis and stability of buckwheat cell wall and membrane components, thereby indirectly regulating the metabolism of auxin and lignin, contributing to the growth of the seedlings. However, either insufficient or excessive concentration of boron fertilizer was found to be unfavorable for the growth of Suqiao 1 and 1412–69 seedlings. Our results are similar to a previous research (Zhang et al., 2023), in which it was found that either boron deficiency or toxicity are detrimental to the growth of mulberry trees. The optimal concentration of boron that we established in the present study differs from what has been reported in other studies, possibly due to different requirements depending on species and variety as well as abiotic factors such as soil conditions. Accordingly, the effect of foliar application of boron is expected to differ depending on the crop type. The exact reasons for this need further investigation.

The SPAD value is an effective predictor of crop yield (Kim and Kim, 2024). Boron has significant effects on the chloroplast structure, chlorophyll synthesis, and stability in plant leaves. Boron deficiency can result in damage to the structure and function of plant leaves, ultimately leading to a reduction in the content of photosynthetic pigments (Song et al., 2022). Li et al. (2023) constructed a molecular mechanism model for the boron deficiency response in tomato through physiological, biochemical, and transcriptome analyses. The authors of that study found that boron deficiency in tomato increased the accumulation of copper, manganese, and iron, thereby maintaining chlorophyll content and photosynthetic efficiency during the early stages of stress (Li et al., 2023). In the present study, we found that the chlorophyll SPAD values of the two varieties of buckwheat seedlings showed a trend of first increasing and then decreasing with increasing boron fertilizer concentration. The change in chlorophyll SPAD values in 1412–69 was greater than that of Suqiao 1. Therefore, spraying appropriate concentrations of boron fertilizer on buckwheat growing on boron-deficient soil during the jointing stage can enhance leaf SPAD values, increase chlorophyll content, and improve photosynthetic rate in both varieties.

### 4.2 Concentration-dependent effect of boron fertilizer on the antioxidant enzyme activity of buckwheat

Boron can regulate the activity of some enzymes in plants, activating or inhibiting them (Qin et al., 2022). SOD, CAT and glutathione peroxidase (GPx) are enzymatic antioxidant systems that regulate the homeostasis of ROS in organisms (Jomova et al., 2024). Under normal conditions, the rate of ROS production and scavenging in plant metabolism is in a dynamic equilibrium. When plants are stressed by an adverse external environment, the production rate of ROS will be higher than the clearance rate, causing oxidative damage to plants. When boron is lacking in the plant, the balance between ROS production and clearance is disrupted, leading to the accumulation of a large amount of ROS between cells and causing oxidative damage to the plant body. Suitable supplementation of boron can reduce ROS content, inhibit membrane peroxidation, and protect plants from oxidative damage (Chen et al., 2024; Wang et al., 2024b). Therefore, it is crucial to supplement boron to plants growing on boron deficient soil. In the present study, as the mass concentration of boron fertilizer solution increased, the level of activity of SOD, CAT, and POD in the seedlings of two buckwheat varieties showed a trend of first increasing and then decreasing. When the mass concentration of boron fertilizer solution was 48 mg/L, the activity of SOD, CAT, and POD in Suqiao 1 seedlings were the highest. When the mass concentration of boron fertilizer solution was 24 mg/L, the activity of SOD, CAT, and POD of 1412–69 seedlings reached a maximum. A previous study (Zhang et al., 2023) stated that boron deficiency and toxicity resulted in decreased CAT and SOD activity and increased POD activity in mulberry trees. Another study (Huo et al., 2022) found that boron toxicity reduced CAT activity in sugar beets while increasing POD and SOD activities and MDA accumulation. These reports share similarities with our research. Furthermore, we demonstrated that MDA content in both buckwheat varieties exhibited an initial decrease followed by an increase with rising boron concentrations, inversely correlating with SOD, CAT, and POD trends. This result suggests that an appropriate concentration could enhance SOD-mediated oxygen free radical scavenging. However, excessive concentrations reduced seedling respiration rates, decreased stress-resistant enzyme activity, elevated MDA levels, and ultimately impaired seedling growth quality.

### 4.3 Concentration-dependent effect of boron on the yield and grain quality of buckwheat

Boron is one of the indispensable trace elements in the process of plant growth. An appropriate amount of boron fertilizer can promote root growth, improve plant photosynthesis, be beneficial to the synthesis and transport of carbohydrates, enhance the resistance of crops to disease, and improve crop yield. Boron deficiency during crop growth can inhibit its growth and development, resulting in decreased yield and quality (Arrobas et al., 2023). In the present study we found that spraying boron fertilizer during the budding stage significantly increased the grain weight per plant, thousand grain weight, and plot yield of two buckwheat varieties. Moreover, the application of 48 mg/L boron fertilizer can effectively increase the yield of Suqiao 1, whereas the application of 24 mg/L boron fertilizer can effectively increase the yield of 1412-69. Similarly, the application of appropriate amounts of boron fertilizer can increase the yield of crops such as tomato (Haleema et al., 2024), soybean (Galeriani et al., 2022), and cotton (da Silva Liber Lopes et al., 2023) to varying degrees. Due to the ability of boron to enhance crop root vitality, spraying an appropriate amount of boron fertilizer on the leaves of crops grown in boron-deficient soil can increase chlorophyll content, photosynthesis efficiency, and seed setting rate, ultimately increasing yield (Galeriani et al., 2022). It is worth noting that when the concentration of boron fertilizer solution is too high, the yield of both varieties of buckwheat showed a downward trend, indicating that buckwheat is very sensitive to the demand for boron fertilizer. Spraying an appropriate amount of boron fertilizer has a yield increasing effect on buckwheat. However, when the amount of boron fertilizer applied exceeds a certain range, a negative effect occurs, reducing yield.

Buckwheat grains contain various nutrients such as protein, polysaccharides, lipids, rutin, trace elements, and macroelements (Vieites-Álvarez et al., 2024). These nutrients seem to be important for the quality of buckwheat-based products. The main bioactive compounds identified in buckwheat are rutin, quercetin, isorcetin, D-luteinol, resveratrol, and vitexin, which are the main components of its pharmacological properties (Raina et al., 2024). Moreover, flavonoids play an important role in human nutrition and can prevent a variety of chronic diseases. The increase of flavonoid content can greatly improve the functional role of buckwheat products, increase the market demand for buckwheat products, and promote the enthusiasm of farmers (Kreft et al., 2022b). Quercetin and rutin have antioxidant, anti-inflammatory, antiviral and antibacterial effects, and exhibit preventive and therapeutic effects on cardiovascular and cerebrovascular diseases, malignant tumors, hyperglycemia, brain neurodegeneration, joint inflammation and eye diseases (Mirza et al., 2023; Wang et al., 2024a). A previous study demonstrated that quercetin attenuates high-fat diet-induced obesity, while both rutin and quercetin modulate specific gut microbiota structures (Peng et al., 2020). The results of this experiment showed that the contents of protein, starch, soluble sugar, total flavonoids, quercetin and rutin in the grain of two buckwheat varieties first increased and then decreased after foliar spraying boron fertilizer. In conclusion, foliar spraying with appropriate concentration of boron fertilizer solution could improve the post-harvest grain quality of Suqiao 1 and 1412-69, but excessive concentration of boron fertilizer solution would have the opposite effect. Additionally, boron fertilization significantly improves the mineral nutrient status of plants and regulates the uptake and utilization of other nutrients (Long and Peng, 2023). For example, in maize, boron enhances nitrogen use efficiency by promoting the synthesis of nitrate reductase (Fuertes-Mendizábal et al., 2020). Moreover, boron application substantially increased boron accumulation in sugar beet roots, manifested as reduced amino nitrogen and sodium contents alongside an increase in sucrose content (Wu et al., 2024). Research has also demonstrated that the interaction between boron and phosphorus in boron-deficient soils affects the growth and yield of rapeseed, where an imbalance in boron and phosphorus supply exacerbates B-deficiency symptoms (Zhao et al., 2025). These findings provide valuable insights for our subsequent research. In our forthcoming work, we will include a broader range of buckwheat varieties to analyze the effects of different concentrations of boron fertilizer on other nutrients in buckwheat.

Although the amount of boron fertilizer used in production is small, the effect is significant. When applying boron fertilizer, it is necessary to understand the optimal fertilization amount of boron fertilizer. Both excessive and insufficient boron have inhibitory effects on crop growth, affecting crop yield and quality. Appropriate boron fertilizer is of great significance to improve crop yield and quality. A study has revealed that boron supplementation in soybeans can decrease oxidative stress indicators, including MDA and hydrogen peroxide. Additionally, under salt stress conditions, boron supplementation has been shown to increase the concentrations of K^+^ and Ca^2+^, enhance the activities of CAT, SOD, POD, and secondary metabolites. These results indicate that boron can ameliorate oxidative damage caused by salinity in soybean plants by modulating antioxidant defenses, secondary metabolites, and maintaining ionic homeostasis (Tanveer et al., 2025). Furthermore, lipid peroxidation in the leaves of Highbush Blueberry plants treated with high concentrations of boron (800 mg/L) gradually increased over time (Reyes-Díaz et al., 2024). In another study it was found that, under boron stress, the growth rate of maize leaves declined, with gas exchange being restricted and high levels of hydrogen peroxide accumulation and lipid peroxidation occurring. These findings indicate that high concentrations of boron exacerbate oxidative stress, subsequently inhibiting plant growth (Koyukan et al., 2025).This study explored the effects of different concentrations of boron fertilizer on seed germination, seedling growth, physiological indices, yield and grain quality of buckwheat. The mechanism of deeper physiological and biochemical reactions and the reasons for the inhibition of seed germination, seedling growth, yield and grain quality by high concentrations of boron fertilizer solution require further investigation.

In this study, the difference in optimal boron concentration requirements between the two buckwheat varieties, Suqiao 1 (48-mg/L) and 1412-69 (24-mg/L), could be related to genetic and physiological mechanisms. Firstly, differential expression of boron transporter genes may exist between varieties. For example, Suqiao 1 may exhibit higher expression of high-affinity boron transporters genes, allowing it to tolerate and adapt to higher boron concentrations, whereas 1412–69 may rely primarily on low-affinity transport system. Secondly, the enzymes involved in boron metabolism could differ in their sensitivity to boron concentrations between the two varieties. Suqiao 1 may require higher boron concentrations to activate key metabolic pathways; in contrast, the enzyme system of 1412-69 may be adapted to low-concentration environments. Thirdly, Suqiao 1 possesses more developed root hairs, expanding the absorption surface area and preventing boron toxicity even at high concentrations. In contrast, the shallow root system of 1412–69 may be suited to rapid boron absorption at low concentrations. Therefore, future studies should further explore the differential optimal boron requirements between Suqiao 1 and 1412-69.

The results of this experiment indicated that different concentrations of boron fertilizer solutions had different effects on buckwheat seed germination, seedling growth, physiological indicators, yield, and grain quality. An appropriate concentration of boron fertilizer could promote seed germination, improve seedling growth, enhance yield and grain quality of buckwheat, while both excess and deficiency of boron inhibited growth, reducing yield and grain quality. Based on the above analysis, 48 and 24 mg/L boron fertilizer solution are recommended for the actual production of Suqiao 1 and 1412-69, respectively.

## Data Availability

The raw data supporting the conclusions of this article will be made available by the authors, without undue reservation.
